# ﻿Physical chromosomal mapping of major ribosomal genes in 15 ant species with a review of hypotheses regarding evolution of the number and position of NORs in ants

**DOI:** 10.3897/compcytogen.18.125235

**Published:** 2024-06-26

**Authors:** Monique Telcia dos Santos Damasceno, Gisele Amaro Teixeira, Paulo Castro Ferreira, Rodrigo Batista Lod, Luísa Antônia Campos Barros, Hilton Jeferson Alves Cardoso de Aguiar

**Affiliations:** 1 Universidade Federal do Amapá, Campus Binacional – Oiapoque, n°3051, Bairro Universidade, Oiapoque, Amapá, 68980-000, Brazil Universidade Federal do Amapá Oiapoque Brazil; 2 Programa de Pós-graduação em Biodiversidade Tropical, Universidade Federal do Amapá, Campus Marco Zero do Equador, Macapá, Amapá, 68.903-419, Brazil Universidade Federal do Amapá Macapá Brazil

**Keywords:** Formicidae, FISH, karyotype, molecular cytogenetics, rDNA sites

## Abstract

Recently, hypotheses regarding the evolutionary patterns of ribosomal genes in ant chromosomes have been under discussion. One of these hypotheses proposes a relationship between chromosomal location and the number of rDNA sites, suggesting that terminal locations facilitate the dispersion of rDNA clusters through ectopic recombination during meiosis, while intrachromosomal locations restrict them to a single chromosome pair. Another hypothesis suggests that the multiplication of rDNA sites could be associated with an increase in the chromosome number in Hymenoptera due to chromosomal fissions. In this study, we physically mapped rDNA sites in 15 new ant species and also reviewed data on rDNA available since the revision by Teixeira et al. (2021a). Our objectives were to investigate whether the new data confirm the relationship between chromosomal location and the number of rDNA sites, and whether the increase in the chromosome number is significant in the dispersion of rDNA clusters in ant karyotypes. Combining our new data with all information on ant cytogenetics published after 2021, 40 new species and nine new genera were assembled. Most species exhibited intrachromosomal rDNA sites on a single chromosome pair, while three species showed these genes in terminal regions of multiple chromosome pairs. On one hand, the hypothesis that the chromosomal location of rDNA clusters may facilitate the dispersion of rDNA sites in the ant genome, as previously discussed, was strengthened, but, on the other hand, the hypothesis of chromosomal fission as the main mechanism for dispersion of ribosomal genes in ants is likely to be refuted. Furthermore, in certain genera, the location of rDNA sites remained similar among the species studied, whereas in others, the distribution of these genes showed significant variation between species, suggesting a more dynamic chromosomal evolution.

## ﻿Introduction

In Formicidae, molecular cytogenetic studies involving fluorescence *in situ* hybridization (FISH) for physical mapping of major ribosomal genes, 45S ribosomal DNA (rDNA), here referred to as rDNA clusters, were first conducted in Australian ants of the genus *Myrmecia* Fabricius, 1804 (Imai et al. 1992; Hirai et al. 1994, 1996). In recent years, FISH has been widely employed in several ant species, particularly in the Neotropical region (Santos et al. 2016; Aguiar et al. 2017; Micolino et al. 2019, 2022; Barros et al. 2021a, b, 2022a, b; Murakami et al. 2021; Silveira 2022; Teixeira et al. 2022, 2023; Jacintho et al. 2023). These molecular cytogenetic studies have provided valuable insights into various biological aspects of these insects, including evolution, taxonomy, and reproduction.

For instance, the physical mapping of ribosomal genes in certain ant genera has enabled the proposal of chromosomal rearrangements during their karyotypic evolution, such as the occurrence of inversions in *Myrmecia* (Hirai et al. 1996), *Dolichoderus* Lund, 1831 (Santos et al. 2016), *Mycetophylax* Emery, 1913 (Micolino et al. 2019), and *Acromyrmex* Mayr, 1865 (Barros et al. 2016; Teixeira et al. 2021a). Additionally, chromosomal polymorphisms involving the rDNA clusters, with homozygous and heterozygous karyotypes, which may arise from duplications/deletions due to unequal crossing-over or the formation of extrachromosomal circular DNA (eccDNA) have been observed in *Gnamptogenysregularis* Mayr, 1870 (Teixeira et al. 2020a) and *Odontomachusbauri* Emery, 1892 (Teixeira et al. 2021a). These may arise from duplications/deletions due to unequal crossing-over or the formation of extrachromosomal circular DNA (eccDNA) in these species. EccDNA can replicate via a rolling circle mechanism and then either get reintegrated into the genome or deleted from it, respectively causing duplications or deletions of these sequences (Teixeira et al. 2020a, 2021a).

Regarding ant taxonomy, mapping the chromosomal distribution of rDNA clusters has been important in helping to delimit specific boundaries between taxa, as is the case of the ants *Camponotusrenggeri* Emery, 1894 and *Camponotusrufipes* (Fabricius, 1775) (Aguiar et al. 2017). These two species were subjects of discussion regarding taxonomic synonymization. However, the number of chromosomes bearing the rDNA clusters differs between them, with *C.rufipes* possessing one pair and *C.renggeri* possessing two pairs, a hereditary characteristic capable of distinguishing these two *Camponotus* Mayr, 1861 species (Aguiar et al. 2017). Additionally, *C.rufipes* and *C.renggeri* differ in ecological, molecular, and behavioral traits, and this further confirms their status as valid species (Ronque et al. 2015).

Furthermore, cytogenetic data, including the chromosome location of rDNA sites, in the fungus-growing ant *Mycocepurussmithii* (Forel, 1893), have contributed to enhancing the understanding of cytological mechanisms associated with thelytokous parthenogenesis in this species (Barros et al. 2022a). Karyotypic variations were observed in the asexual population (2n=9, 10, and 11) with a decay of the diploid structure in the absence of meiosis and genetic recombination, whereas in the sexual population, the karyotype remained stable (2n=14) with appropriate homologous pairing. The data mapping of rDNA sites in *M.smithii* shows a single chromosome pair bearing these genes in the sexual population and in the karyomorphs 2n=9 and 2n=11 of the asexual population, supporting the idea that asexual individuals are indeed diploids. However, these data demonstrate the decay of the diploid structure, particularly in the 2n=11 karyomorph, in which there is a variation in size between the homologs of the pair bearing rDNA sites (Barros et al. 2022a).

Recently, based on new and previously published data regarding the chromosomal mapping of ribosomal genes from 63 species, 19 genera and six subfamilies of ants, Teixeira et al. (2021a) proposed important insights into the general patterns of these genes in ant chromosomes. These authors showed that rDNA clusters have a non-random distribution within the ant genome in which there is a relationship between chromosomal location and the number of rDNA sites. Most ant species have a single intrachromosomal (pericentromeric/interstitial) rDNA site, whereas species with multiple rDNA sites have these genes located in the terminal regions. Based on Hirai’s model (2020), Teixeira et al. (2021a) argued that the terminal location of rDNA sites in ants would facilitate association with other non-homologous chromosome terminal sequences during the meiotic bouquet, forming affinity systems, which would lead to the occurrence of ectopic recombination and dispersion of rDNA clusters to other chromosomes. However, the intrachromosomal location of rDNA sites would hinder interaction with other chromosomes, restricting these genes to a single chromosome pair.

Alternatively, Menezes et al. (2021) proposed that the multiplication of rDNA sites could be linked to an increase of the chromosome number in most groups of Hymenoptera (ants, wasps and bees), suggesting that chromosomal fissions play a pivotal role in the dispersal of rDNA clusters in the karyotypes of these insects.

Despite notable advances in molecular cytogenetic data in ants, entire genera and even subfamilies have not yet been studied in this respect. Thus, in this study, we performed chromosomal mapping of ribosomal genes through FISH in 15 new ant species belonging to 9 genera, and also reviewed molecular cytogenetic data involving rDNA sites available since the paper by Teixeira et al. (2021a) was published. Our goal was to verify whether the chromosomal distribution of ribosomal genes in these ant species follows a relationship between the chromosomal location and the number of rDNA sites, and whether the increase in the chromosome number is significant in the dispersion of these genes in ant karyotypes.

## ﻿Materials and methods

Field campaigns to collect ant colonies were performed in French Guiana and Brazil in regions of Amazonian and Atlantic rainforests (Table 1) from the following locations: Campus Agronomique, Kourou (5.17312°N, 52.65480°W) and Petit Saut route (5.13051°N, 52.94385°W), both in French Guiana; Oiapoque, Amapá State (3.84151°N, 51.84112°W) and Viçosa, Minas Gerais State (20.75696°S, 42.87314°W), both in Brazil. Sampling license in Brazil was provided by the Instituto Chico Mendes de Conservação da Biodiversidade (ICMBio) (SISBIO accession numbers 87049-1). Adult specimens were deposited in the Coleção Entomológica do Laboratório de Coleoptera (CELC), at the Universidade Federal de Viçosa (UFV), Viçosa, Brazil.

**Table 1.** Ant species in which chromosomal mapping of rRNA genes was performed in this study, their respective localities and Brazilian biomes, and diploid chromosome numbers.

Ant species	Localities	Brazilian biomes	Chromosome numbers
**Dolichoderinae**			
*Aztecaandreae*	Petit Saut route, French Guiana	Amazonian rainforest	2n=28
**Formicinae**			
*Brachymyrmexadmotus*	Viçosa, MG, Brazil	Atlantic rainforest	2n=18
*Brachymyrmexheeri*	Oiapoque, AP, Brazil	Amazonian rainforest	2n=18
*Camponotuscameroni*	Viçosa, MG, Brazil	Atlantic rainforest	2n=36
*Nylanderia* sp.	Viçosa, MG, Brazil	Atlantic rainforest	2n=30
**Myrmicinae**			
*Cephalotescordatus*	Oiapoque, AP, Brazil	Amazonian rainforest	2n=24
*Cephalotesminutus*	Kourou, French Guiana	Amazonian rainforest	2n=44
*Cyphomyrmexlaevigatus*	Oiapoque, AP, Brazil	Amazonian rainforest	2n=14
Megalomyrmexaff.incisus	Oiapoque, AP, Brazil	Amazonian rainforest	2n=46
*Pheidolejelskii*	Oiapoque, AP, Brazil	Amazonian rainforest	2n=20
*Pheidolevorax*	Oiapoque, AP, Brazil	Amazonian rainforest	2n=42
*Solenopsissaevissima*	Viçosa, MG, Brazil	Atlantic rainforest	2n=32
*Strumigenysschulzi*	Oiapoque, AP, Brazil	Amazonian rainforest	2n=18
**Ponerinae**			
*Neoponeraunidentata*	Oiapoque, AP, Brazil	Amazonian rainforest	2n=12
*Pseudoponerastigma*	Oiapoque, AP, Brazil	Amazonian rainforest	2n=14

Brazilian states: MG- Minas Gerais; AP - Amapá.

In addition, for comparative purposes, we performed a survey of molecular cytogenetic data involving chromosomal mapping of ribosomal genes through FISH in ants since the last review by Teixeira et al. (2021a). The cytogenetic data are shown in Table 2 and the following traits were considered for each species: general number of chromosomes, number of chromosomes bearing rDNA clusters, and location of rDNA clusters on chromosomes.

**Table 2.** Summary of the available molecular cytogenetic data, including this study and published data after the revision by Teixeira et al. (2021a), concerning major rDNA clusters detected by FISH in ants.

Species	2n	rDNA cluster location		References
		Chromosome pair	Chromosome region	
**Dolichoderinae**				
*Aztecaandreae*	28	2^nd^ sm	short arm	**This study**
*Technomyrmexvitiensis*	16	m	pericentromeric	Barros et al. (2022b)
**Formicinae**				
*Brachymyrmexadmotus*	18	8^th^ m	pericentromeric	**This study**
*Brachymyrmexheeri*	18	8^th^ m	pericentromeric	**This study**
*Camponotuscameroni*	32	4^th^ sm, 6^th^ sm, 7^th^ st and 8^th^ st	short arm	**This study**
*Nylanderia* sp.	30	10^th^ a	pericentromeric	**This study**
**Myrmicinae**				
*Acromyrmexameliae*	36	1^st^ st	terminal	Barros et al. (2021b)
*Acromyrmexbalzani*	38	1^st^ st	short arm	Barros et al. (in press)
*Acromyrmexbrunneus*	38	1^st^ st	terminal	Barros et al. (in press)
*Acromyrmexlaticeps*	38	1^st^ st	terminal	Barros et al. (in press)
*Acromyrmexsubterraneus*	38	1^st^ st	terminal	Barros et al. (in press)
*Amoimyrmexbruchi*	22	2^nd^ m	pericentromeric	Micolino et al. (2022)
*Amoimyrmexsilvestrii*	22	2^nd^ m	pericentromeric	Micolino et al. (2022)
*Attacephalotes*	22	4^th^ m	interstitial	Teixeira et al. (2022)
*Cephalotescordatus*	24	1^st^ sm	pericentromeric	**This study**
*Cephalotesminutus*	44	7^th^ sm	short arm	**This study**
Crematogasteraff.erecta	28	3^rd^ m	pericentromeric	Silveira (2022)
*Crematogastererecta* cytotype I	22	2^nd^ sm	interstitial	Silveira (2022)
*Crematogastererecta* cytotype II	22	3^rd^ m	pericentromeric	Silveira (2022)
*Crematogasterlimata*	38	1^st^ m	pericentromeric	Silveira (2022)
*Crematogaster* sp.	38	5^th^ m	interstitial	Silveira (2022)
*Crematogastertenuicula*	38	5^th^ m	interstitial	Silveira (2022)
*Cyphomyrmexlaevigatus*	14	5^th^ m	pericentromeric	**This study**
*Cyphomyrmexrimosus*	22	3^rd^ m	pericentromeric	Teixeira et al. (2023)
*Cyphomyrmextransversus*	18	2^nd^ m	pericentromeric	Teixeira et al. (2021b)
*Eurhopalothrixreichenspergeri*	16	2^nd^ m	terminal	Jacintho et al. (2023)
Megalomyrmexaff.incisus	46	4^th^ m	pericentromeric	**This study**
*Mycetomoelleriusrelictus*	20	5^th^ m	interstitial	Teixeira et al. (2021b)
*Mycocepurussmithii*	9	1^st^ sm	interstitial	Barros et al. (2022a)
	11	1^st^ sm	interstitial	
	14	1^st^ sm	interstitial	
*Pheidolejelskii*	20	1^st^ m	pericentromeric	**This study**
*Pheidolevorax*	42	1^st^ st	pericentromeric	**This study**
*Solenopsisinvicta* (native population from Argentina)	32	two chromosome pairs	short arm	Murakami et al. (2021)
*Solenopsissaevissima*	32	1^st^ sm and 5^th^ sm	short arm	**This study**
*Strumigenyscrassicornis*	26	3^rd^ m	interstitial	Jacintho (2023)
*Strumigenysdenticulata*	18	2^nd^ m	pericentromeric	Jacintho (2023)
*Strumigenyslouisianae*	4	1^st^ m	interstitial	Jacintho (2023)
	20	2^nd^ m	pericentromeric	Barros et al. (2021b)
	26	4^th^ m	interstitial	Jacintho (2023)
*Strumigenysschulzi*	18	3^rd^ m	pericentromeric	**This study**
Strumigenysaff.stenotes	16	2^nd^ m	interstitial	Jacintho (2023)
*Strumigenyssubedentata*	18	3^rd^ m	pericentromeric	Jacintho (2023)
**Ponerinae**				
*Neoponeraunidentata*	12	6^th^ m	pericentromeric	**This study**
*Pseudoponerastigma*	14	3^rd^ m	pericentromeric	**This study**

Chromosomal classification: m – metacentric; sm – submetacentric; st – subtelocentric.

For cytogenetic analysis, mitotic metaphase chromosomes were obtained from the cerebral ganglia of larvae after meconium elimination according to the methods described by Imai et al. (1988). The 18S rDNA probes were amplified via polymerase chain reaction (PCR) using primers 18SF1 (5`-TCATATGCTTGTCTCAAAG-3`) and 18SR1.1 (3`-TCTAATTTTTTCAAAGTAAACG-5`) designed for *Meliponaquinquefasciata* Lepeletier, 1836 (Pereira 2006) in the genomic DNA from the ant *Camponotusrufipes*. Gene amplification was performed following Pereira (2006). The probes were labeled with digoxigenin-11-dUTP using Dig-Nick-Translation Mix (Roche Applied Science, Mannheim, Germany), and the FISH signals were detected with anti-digoxigenin-rhodamine (Roche Applied Science), following the manufacturer’s protocol.

The rDNA sites were mapped on the chromosomes of Neotropical ant species using FISH according to Pinkel et al. (1986) with modifications described in Teixeira et al. (2021a): the slides were treated with RNase A (100 μg/ml) and kept in a moist chamber at 37 °C for 1 h. After that, they were washed in 2×SSC for 5 min, incubated in 5 μg/ml pepsin in 0.01 N HCl for 10 min, washed in 1× PBS for 5 min, and dehydrated in 50%, 70% and 100% alcohol series for 2 min each. After this pretreatment, metaphase chromosomes were denatured in 70% formamide/2×SSC at 75 °C for 5 min, and 20 μl of hybridization mix including 200 ng of labeled probe, 2×SSC, 50% formamide, and 10% dextran sulfate was denatured for 10 min at 85 °C and added on preparations. The slides were kept in a moist chamber up to 37 °C overnight. Then, the slides were washed in 2×SSC for 5 minutes; the detection solution including anti-digoxigenin-rhodamine was added on slides that were kept in a moist chamber at 37 °C for 1 h. The slides were washed three times in 4×SSC/Tween 20 (4×SSC, 0.05% [v/v] Tween 20) and dehydrated in an alcohol series. Finally, counterstaining with DAPI (DAPI Fluoroshield, Sigma Aldrich) was performed.

Slides subjected to FISH with the 18S rDNA probes were photographed using an epifluorescence microscope Olympus BX60 attached to an Olympus DP23M camera, and CellSens image capture software, using the filters WG (510–550 nm) for the rhodamine, and WU (330–385 nm) for DAPI. Images of the chromosomes were arranged using Adobe Photoshop® CS6. At least 20 metaphases for each species were analyzed to determine the FISH patterns.

## ﻿Results

**Figure 1.**
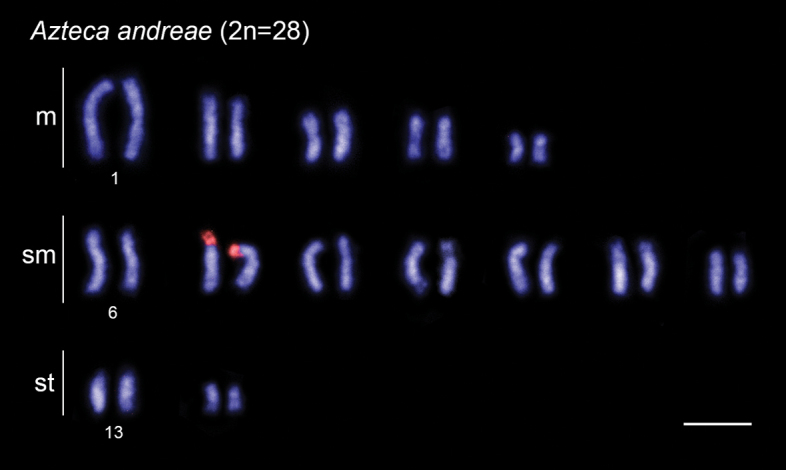
FISH with 18S rDNA probe (red signals) performed in ant *Aztecaandreae* (Dolichoderinae). Scale bar: 5 µm.

**Figure 2.**
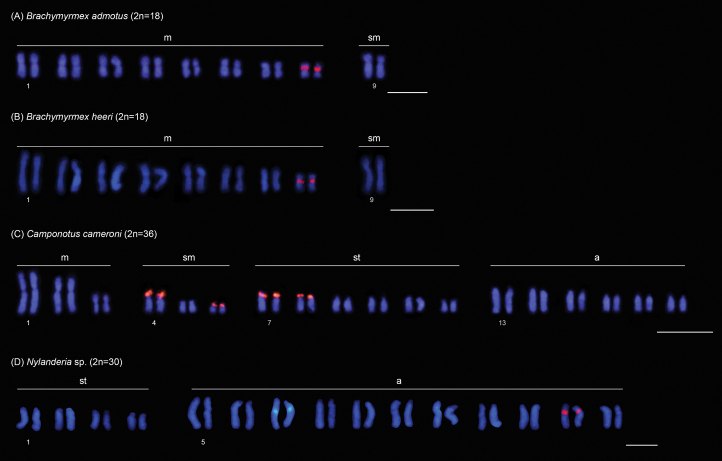
FISH with 18S rDNA probe (red signals) performed in different ant species of the subfamily Formicinae. Scale bars: 5 µm.

**Figure 3.**
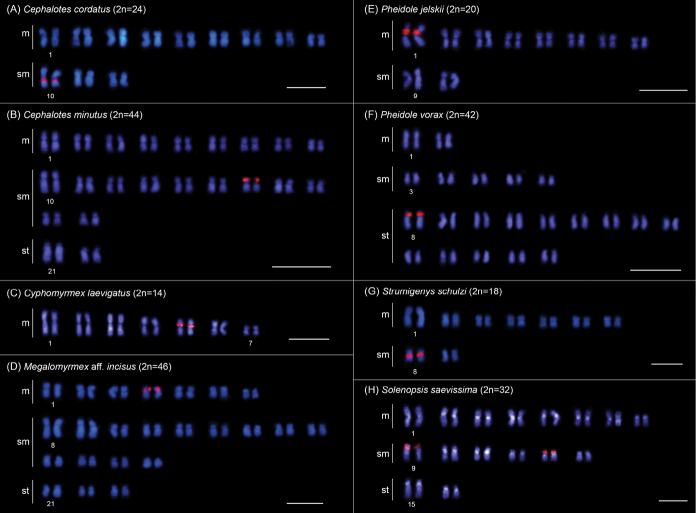
FISH with 18S rDNA probe (red signals) performed in different ant species of the subfamily Myrmicinae. Scale bars: 5 µm.

**Figure 4.**
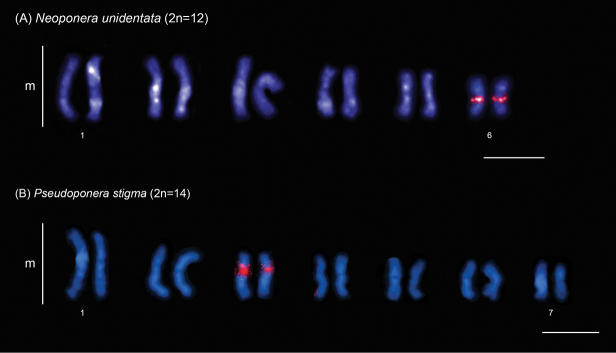
FISH with 18S rDNA probe (red signals) performed in different ant species of the subfamily Ponerinae. Scale bars: 5 µm.

In this study, we physically mapped rDNA genes in the karyotypes of 15 species from 9 genera and 4 subfamilies (data for six genera have not been previously published) (Table 1). Among these species, 13 exhibit a single chromosomal pair bearing rDNA clusters, which are located in the pericentromeric region: in *Brachymyrmexadmotus* Mayr, 1887 (Fig. 2A), *B.heeri* Forel, 1874 (Fig. 2B), *Nylanderia* sp. (Fig. 2D), *Cephalotescordatus* (Smith, 1853) (Fig. 3A), *Cyphomyrmexlaevigatus* Weber, 1938 (Fig. 3B), Megalomyrmexaff.incisus Smith, 1947 (Fig. 3D), *Pheidolejelskii* Mayr, 1884 (Fig. 3E), *P.vorax* (Fabricius, 1804) (Fig. 3F), *Strumigenysschulzi* Emery, 1894 (Fig. 3G), *Neoponeraunidentata* (Mayr, 1862) (Fig. 4A), and *Pseudoponerastigma* (Fabricius, 1804) (Fig. 4B). In *Aztecaandreae* Guerrero, Delabie et Dejean, 2010 (Fig. 1) and *Cephalotesminutus* (Fabricius, 1804) (Fig. 3C), the rDNA sites are located on the short arms. Furthermore, two species showed rDNA clusters on the short arms of more than one chromosome pair: *Camponotuscameroni* Forel, 1892 in two submetacentric and two subtelocentric pairs (Fig. 2C) and *Solenopsissaevissima* (Smith, 1855) in two submetacentric pairs (Fig. 3H).

Chromosome mapping data from rDNA sites, which have been available since the review conducted by Teixeira et al. (2021a), encompassing 25 species across 10 genera (including three new genera), were reviewed (Table 2). This new information focused on ants from the Neotropics. However, some invasive populations of *Solenopsisinvicta* Buren, 1972 from eastern Asia were also investigated. In most taxa, a single chromosomal pair bearing rDNA sites, located in the pericentromeric or interstitial regions, was observed. However, populations of *S.invicta* exhibited multiple rDNA terminal sites: the native population from Argentina had two chromosome pairs carrying rDNA clusters, while invasive populations from Argentina, United States, and Taiwan showed large variation on this pattern, from 1 to 11 chromosomes carrying rDNA sites depending on the ploidy (Table 2).

## ﻿Discussion

Molecular cytogenetic data, which involve chromosomal mapping of rDNA clusters in ants, are now available for 103 species/subspecies, 28 genera and 6 subfamilies (this study, Table 2; reviewed in Teixeira et al. 2021a). Considering the number of chromosome pairs bearing rDNA clusters observed among the ant taxa analyzed since the review by Teixeira et al. (2021a) was published, 36 of them showed only a single pair bearing these genes while three species, namely *S.saevissima* and *S.invicta*, had two pairs, and *C.cameroni* had four pairs (Table 2). These patterns indicate that a diploid genome with a single chromosome pair carrying ribosomal genes should be considered an ancestral feature in Formicidae, as previously discussed by Teixeira et al. (2021a), and later suggested for the Hymenoptera in general (Menezes et al. 2021).

The rDNA physical mapping also strongly reinforces the relationship between the number of rDNA sites and their location discussed by Teixeira et al. (2021a) since the species *C.cameroni*, *S.saevissima*, and *S.invicta* have multiple rDNA sites located on short arms (including terminal regions) of the chromosomes while most other species studied show a single intrachromosomal rDNA site (Table 2). Based on the Hirai’s model (2020), terminal location of rDNA sites in ants would facilitate association with other non-homologous chromosome terminal sequences during the meiotic bouquet, forming affinity systems, which would lead to the occurrence of ectopic recombination and dispersion of rDNA clusters to other chromosomes. Hirai’s (2020) model for the dispersion of rDNA clusters in karyotypes does not address the presence of haploid males (haplodiploid reproductive system), such as those found in ants. We hypothesize that males could inherit rearrangements involved in the dispersal of rDNA clusters that occurred in the queens’ genomes. Due to the haploid nature of hymenopteran males, the occurrence of ectopic recombination is restricted to females which are diploid. Therefore, it is possible that the evolution of repetitive DNA sequences (such as rDNA clusters), through the mechanisms described by Hirai (2020) may be slower in Hymenoptera when compared to other insect orders that reproduce in diplodiploid fashion.

Furthermore, our results seem to refute the hypothesis of chromosomal fission as the main mechanism for dispersion of ribosomal genes in ants proposed by Menezes et al. (2021) since: (i) species with the same chromosome number show differences in the number of rDNA sites, such as in *Solenopsisgeminata* (Fabricius, 1804) (2n=32; one pair with rDNA), *S.saevissima* (2n=32; two pairs with rDNA) and the native population of *S.invicta* from Argentina (2n=32; two pairs with rDNA), in addition to *C.rufipes* (2n=40; one pair with rDNA) and *C.renggeri* (2n=40; two pairs with rDNA); (ii) in *Camponotus* Mayr, 1861, *C.cameroni* has 2n=36 chromosomes and four pairs bearing rDNA sites compared to other *Camponotus* species with 2n=40 chromosomes and only one or two pairs with rDNA clusters; (iii) in the fungus-growing ant *M.smithii*, asexual populations had a certain degree of relaxed chromosome stability (2n=9 and 11) when compared to sexual populations (2n=14) as discussed by Barros et al. (2022a), and still both populations had a single chromosome pair bearing the intrachromosomal rDNA sites; and (iv) in several ant genera studied, a wide variation in chromosome number is observed between species that present only a single pair with rDNA sites, for example, *Cephalotes* Latreille, 1802 (2n=24 to 44), *Pheidole* Westwood, 1839 (2n=20 to 42), and *Strumigenys* Smith, 1860 (2n=4 to 40) (this study, Table 2, reviewed in Teixeira et al. 2021a). As discussed by Hirai (2020), centric fissions could generate chromosomes with very short heterochromatic arms (acrocentrics) and rDNA clusters in terminal/subterminal positions, which could facilitate affinity associations between these genes and other terminal chromosomal sequences promoting dispersion of rDNA sites in the karyotype of ant species.

For several ant genera studied, only single species have any kind of molecular cytogenetic data on rDNA clusters available, as in *Nylanderia* Emery, 1906, *Megalomyrmex* Forel, 1885, and *Neoponera* Emery, 1901 (this study, Table 2, reviewed in Teixeira et al. 2021a), which limits comparisons and in-depth discussion, however, these data are important to start understanding the chromosomal evolution of ribosomal genes in these genera. For other genera, such as *Brachymyrmex* Mayr, 1868, *Camponotus*, *Solenopsis* Westwood, 1840, *Pseudoponera* Emery, 1900, *Strumigenys*, *Cyphomyrmex* Mayr, 1862, *Pheidole*, and *Cephalotes* ribosomal gene mapping data is available for some species (this study, Table 2, reviewed in Teixeira et al. 2021a), which allowed interspecific comparisons and the observation of some karyotypic patterns, as discussed below.

### ﻿rDNA cluster distribution patterns in the subfamily Dolichoderinae

The subfamily Dolichoderinae includes 22 genera and more than 900 species, grouped into four monophyletic tribes: Tapinomini, which is sister to the clade encompassing Bothriomyrmecini, Dolichoderini, and Leptomyrmecini (Ward et al. 2010). Data on the distribution of rDNA clusters in this subfamily are available for two species in Tapinomini and some species in Dolichoderini (reviewed in Teixeira et al. 2021a; Barros et al. 2022b). All these species had only one pair of chromosomes bearing rDNA sites.

In this study, we provide the first data for the arboreal ant genus *Azteca* Forel, 1878 (comprising 84 valid species, Bolton 2024), which is included in the tribe Leptomyrmecini. We performed the chromosomal mapping of rDNA sites through FISH in *A.andreae* (2n=28). However, location data of Nucleolar Organizer Regions (NORs), which include the major ribosomal genes (45S), obtained through Ag-NOR banding, are available for *A.trigona* Emery, 1893 (2n=28) (Cardoso et al. 2012). Although the karyotypes of these two *Azteca* species were organized according to different chromosomal classification systems, it is possible to observe that the data from FISH with rDNA probe in *A.andreae* and the Ag-NOR banding in *A.trigona* were similar with rDNA clusters located in the terminal region of the short arm of a medium-sized chromosome pair (this study, Cardoso et al. 2012). Regarding classical cytogenetic data, six *Azteca* species, including *A.andreae* in this study, had 2n=28 chromosomes, and only *A.alfari* Emery, 1893 had 2n=26 (Mariano et al. 2019; Barros et al. 2021c). Increasing the efforts to physically map rDNA clusters in other *Azteca* species may unveil variations in the location of rDNA sites, similarly to the observed chromosome number in *A.alfari*, thereby enhancing our understanding of karyotypic evolution in this genus.

### ﻿rDNA cluster distribution patterns in the subfamily Formicinae

The subfamily Formicinae encompasses 52 genera and more than 3000 species, grouped into 11 monophyletic tribes, in which Myrmelachistini is sister to the clade that includes all other tribes (Ward et al. 2016). Chromosomal mapping data of rDNA clusters in the subfamily are available for some *Camponotus* species (Camponotini) and *Gigantiopsdestructor* (Fabricius, 1804) (Gigantiopini) (reviewed in Teixeira et al. 2021a). In this study, we present previously unknown data for the basal tribe Myrmelachistini, which includes *Brachymyrmex*, as well as for the tribe Lasiini, which contains *Nylanderia*.

A similar chromosomal distribution pattern of rDNA clusters has been observed in the two *Brachymyrmex* species, which showed these genes located in the pericentromeric region of the smaller metacentric pair. *Brachymyrmex* is composed of 40 species and has a challenging taxonomic history due to some morphological traits such as small body size (3 mm) and superficially similar external morphology among species (Ortiz-Sepulveda et al. 2019). To date, all the three studied taxa had the same karyotype with 2n=18 chromosomes, with rDNA sites mapped to the same regions and chromosomes (this study, Teixeira et al. 2020b). However, these cytogenetic data are limited, and an increase in the number of species studied using classical and molecular cytogenetic methods may reveal the putative presence of any derived lineage with chromosomal distinctions within *Brachymyrmex*.

In contrast, distinct patterns in the number and chromosomal location of rDNA sites were observed among Camponotus species included in the subgenus Myrmobrachys Forel, 1912, varying numbers of pairs bearing rDNA clusters were observed among the studied species: one pair in *C.rufipes* (2n=40), *C.atriceps* (Smith, 1858) (2n=40), and *C.cingulatus* Mayr, 1862 (2n=40), two pairs in *C.renggeri* (2n=40) (Aguiar et al. 2017; Teixeira et al. 2021a), and four pairs in *C.cameroni* (2n=36) (this study). The presence of multiple rDNA sites located in terminal regions in *C.renggeri* and *C.cameroni* may be associated with ectopic recombination, as discussed earlier.

### ﻿rDNA cluster distribution pattern of the subfamily Myrmicinae

The subfamily Myrmicinae comprises 147 genera and over 7000 species, grouped into six monophyletic tribes, with Myrmicini being sister to the clade that includes other five tribes (Ward et al. 2015). This subfamily concentrates the largest number of cytogenetic data concerning the physical location of rDNA clusters, which are available for Attini, Crematogastrini, and Solenopsidini. Nearly all species exhibit only one chromosome pair carrying rDNA sites (reviewed in Teixeira et al. 2021a), except for populations of *S.invicta*, which have multiple terminal rDNA sites (Murakami et al. 2021). In this study, we present the first results for *Cephalotes* (Attini) and *Megalomyrmex* (Solenopsidini).

Within the fire ant genus *Solenopsis* (comprising more than 190 species, Bolton 2024), variations in the number of chromosome pairs carrying rDNA sites were observed. For instance, *S.geminata* (2n=32) exhibits one pair, while *S.saevissima* (2n=32) and the native population of *S.invicta* from Argentina (2n=32) possess two pairs (this study; Teixeira et al. 2021a; Murakami et al. 2021). Additionally, in invasive/established populations of *S.invicta*, notable intraindividual chromosomal variations were observed concerning the number of chromosomes carrying rDNA sites and the ploidy in females and males. For example, females exhibit karyotypes with 1 to 11 chromosomes carrying rDNA sites, depending on their ploidy, while males show haploid to tetraploid karyotypes with 1 to 9 chromosomes carrying rDNA sites (Murakami et al. 2021). These authors suggest hybridization between invasive populations and closely related species, or between genetically distant populations, and/or the use of insecticides to control these ants and other insects as potential causes of these chromosomal variations observed in *S.invicta*. Studies conducted on populations of *S.saevissima* also reveal the presence of polyploid cells in immature stages, but reversal occurs in the final stages of development, suggesting some fitness advantage from the presence of polyploidy in immature stages, necessitating further investigation (Alves-Silva 2016; Andrade et al. 2023).

With many taxonomic issues, the speciose genus *Strumigenys* (with more than 850 species) is subdivided into several groups of species according to morphological traits (Bolton 2000). Chromosomal mapping data of rDNA sites are available for *S.schulzi* (2n=18) of the *schulzi*-group (this study), *S.diabola* Bolton, 2000 (2n=40) of the *S.mandibularis*-group (Teixeira et al. 2021a), *S.denticulata* Mayr, 1887 (2n=18), *S.subedentata* Mayr, 1887 (2n=18), *S.crassicornis* Mayr, 1887 (2n=26) and S.aff.stenotes (Bolton, 2000) (2n=16) from the *gundlachi*-group, and *S.louisianae* Roger, 1863 (2n=4, 20, 26) from the *louisianae*-group (Barros et al. 2021b; Jacintho 2023). These data show a notable variation in the karyotypic distribution pattern of these genes, even in closely related species. For example, *S.denticulata* and *S.subedentata* share the same chromosome number (2n=18); but differ in the chromosome pair bearing these genes, which is the second and third metacentric pair, respectively (Jacintho 2023). Furthermore, in *S.louisianae* three distinct karyotypes, differing in chromosome number and distribution of rDNA clusters, are observed in three different populations, reinforcing the existence of a species complex in this taxon (Barros et al. 2021b; Jacintho 2023). The variations in the location of rDNA sites among species indicate the intensity of the karyotype evolutionary dynamics, encompassing the rDNA regions. A deeper understanding of the evolutionary patterns of these genes in *Strumigenys* could be achieved in a species group context.

The occurrence of chromosomal rearrangements involving the rDNA region during karyotypic evolution in the fungus-growing ant genus *Cyphomyrmex* (comprising 23 valid species, Bolton 2024) has also been suggested (Teixeira et al. 2023). The data obtained in this study reinforce this hypothesis, since *C.laevigatus* Weber, 1938 (2n=14) showed rDNA sites on the short arm of the 4^th^ metacentric pair, differing from the other two *Cyphomyrmex* species previously studied, *C.transversus* Emery, 1894 (2n=18), and *C.rimosus* (Spinola, 1851) (2n=24), where rDNA clusters are located on the short arm of the 2^nd^ pair and the long arm of the 3^rd^ pair, respectively (Teixeira et al. 2021b, 2023).

*Pheidole* is the most speciose ant genus (with more than 1100 species with worldwide distribution), which is subdivided into several species groups based on external morphology (Wilson 2003), and chromosomal mapping data of rDNA sites also show variations among the studied species. Within the *fallax* group, only *P.jelskii* (2n=20) of this study has chromosomal distribution data for rDNA sites, which were located in the pericentromeric region of the largest metacentric pair. Conventional cytogenetics was performed on other species of this group, namely *Pheidolefallax* Mayr, 1870, *P.dentata* Mayr, 1886, *P.desertorum* Wheeler, 1906, *P.hyatti* Emery, 1895, and *P.nitidula* Emery, 1888, and all of them presented 2n=20 chromosomes which is the modal chromosome number among over 70 taxa studied within the genus, including representatives from the Old and New Worlds (reviewed in Lorite and Palomeque 2010 and Cardoso et al. 2018). Classic cytogenetic studies highlight size heteromorphism between the homologs of the largest chromosomal pair in *P.fallax*, *P.nitidula*, and *P.hyatti* (Goñi et al. 1983; Taber and Cokendolpher 1988). Heteromorphisms in the size of rDNA clusters are common in ants, and they can alter the size between homologs of the same chromosome pair (reviewed in Teixeira et al. 2021a). Thus, it is possible to hypothesize that the heteromorphism observed in *P.fallax*, *P.nitidula*, and *P.hyatti* may be related to the difference in size of rDNA clusters. If this is true, the location of the rDNA clusters in the largest chromosome pair in these three species would be similar to that observed in *P.jelskii* and could be the ancestral character in this group.

Furthermore, considering the *tristis* group of *Pheidole*, *P.vorax* of this study had 2n=42 chromosomes, with rDNA clusters located in the pericentromeric region of the short arm of the largest subtelocentric chromosome pair. Another species previously studied and included in the *tristis* group, namely *P.germaini* Emery, 1896, presented 2n=22 chromosomes, with rDNA sites located in the pericentromeric region of the only subtelocentric pair (Teixeira et al. 2021a). Conventional cytogenetics performed on *P.subarmata* Mayr, 1884 (cited as *P.cornutula* Emery, 1890) showed that this species has 2n=20 chromosomes and size heteromorphism between homologs of the largest chromosome pair (Goñi et al. 1983). Based on the discussion above, it is possible that the largest metacentric chromosome pair in *P.subarmata* (2n=20) may carry the rDNA clusters. Considering that this pattern may be the ancestral character in *Pheidole*, since the majority of studied species have 2n=20 chromosomes (reviewed in Lorite and Palomeque 2010 and Cardoso et al. 2018), we hypothesize that the occurrence of chromosomal fissions involving the chromosomal pair carrying rDNA clusters could give rise to the karyotypes in *P.germaini* (2n=22) and *P.vorax* with 2n=42 chromosomes, respectively, and rDNA clusters located in a subtelocentric pair (this study; Teixeira et al. 2021a).

The arboreal ant genus *Cephalotes* comprises 118 species (Bolton 2024) and according to the most recent molecular phylogeny of the genus, which includes 60% of its species, *C.cordatus* occupies a basal position, whereas *C.minutus* has a derived position (Price et al. 2022). The former species showed 2n=24 and rDNA sites located in the pericentromeric region of a larger metacentric pair, while the latter species presented 2n=44 and rDNA sites located in the short arm of a medium-sized submetacentric pair. The occurrence of fissions followed by tandem growth of heterochromatin apparently enhances telomeric stability, and therefore it could explain the increase in the chromosome number from 2n=24 to 2n=44, and inversions could change the chromosomal location of rDNA clusters from the pericentromeric region to the entire short arm.

### ﻿rDNA cluster distribution patterns in the subfamily Ponerinae

The subfamily Ponerinae comprises 50 genera and over 1200 species, divided into two monophyletic tribes: Platythyreini, represented solely by *Platythyrea* Roger, 1863, and Ponerini, which includes all other genera (Schmidt 2013). Chromosomal mapping of rDNA clusters has been performed in some Ponerini species. Most of them exhibit only one chromosomal pair carrying rDNA sites, except for *Dinoponeragigantea* (Perty, 1833), which has multiple terminal rDNA sites (reviewed in Teixeira et al. 2021a). In this study, we provide new data for *Pseudoponera* and the first results on *Neoponera*.

*Pseudoponera* has six valid species (Bolton 2024), and *P.stigma* and *P.gilberti* (Kempf, 1960) are sympatric and share several morphological similarities. There are important examples of the usefulness of molecular cytogenetic data solving taxonomic challenges in ants (Aguiar et al. 2017). *P.stigma* (2n=14) and *P.gilberti* (2n=12) have different karyotypes (Correia et al. 2016), and the patterns of rDNA genes help to distinguish these two *Pseudoponera* species, because *P.stigma* have rDNA sites located in the pericentromeric region of the third metacentric pair (this study), while *P.gilberti* shows these genes located in the interstitial region of the largest metacentric pair (Teixeira et al. 2021a).

## ﻿Conclusions

In summary, the molecular cytogenetic data from this study, as well as those available after the publication of the revision by Teixeira et al. (2021a), describe chromosome patterns for 40 new species and nine new genera. These new data strengthen the hypothesis suggesting that a single rDNA site per haploid genome represents the ancestral condition in ants. Furthermore, the data reinforce the observed non-random chromosomal distribution of ribosomal genes in Formicidae karyotypes, in which the chromosomal location (terminal or intrachromosomal) of these genes possibly influences the dispersion of rDNA sites in the ant genome. In certain genera, the location of rDNA sites in relation to which chromosomal pair carries the rDNA sites and whether it is located on the short or long arm, remained similar among the species studied, however in others, the distribution of these genes exhibited significant variation between species, suggesting a more dynamic chromosomal evolution. The expansion of molecular cytogenetic studies encompassing other ant subfamilies will continue to enhance our understanding of the chromosomal evolution of ribosomal genes in the genomes of these insects.
